# *Yersinia pseudotuberculosis* and *Y. enterocolitica* Infections, FoodNet, 1996–2007

**DOI:** 10.3201/eid1603.091106

**Published:** 2010-03

**Authors:** Cherie Long, Timothy F. Jones, Duc J. Vugia, Joni Scheftel, Nancy Strockbine, Patricia Ryan, Beletshachew Shiferaw, Robert V. Tauxe, L. Hannah Gould

**Affiliations:** Georgia Department of Human Resources, Atlanta, Georgia, USA (C. Long); Atlanta Research and Education Foundation, Atlanta (C. Long); Tennessee Department of Health, Nashville, Tennessee, USA (T.F. Jones); California Department of Public Health, Richmond, California, USA (D.J. Vugia); Minnesota Department of Health, St. Paul, Minnesota, USA (J. Scheftel); Centers for Disease Control and Prevention, Atlanta (N. Strockbine, R.V. Tauxe, L.H. Gould); Maryland Department of Health and Mental Hygiene, Baltimore, Maryland, USA (P. Ryan); Oregon Department of Human Services, Portland, Oregon, USA (B. Shiferaw)

**Keywords:** Yersinia pseudotuberculosis, FoodNet, gastroenteritis, lymphadenitis, Yersinia enterocolitica, zoonoses, bacteria, letter

**To the Editor:** *Yersinia pseudotuberculosis*, a gram-negative zoonotic bacterial pathogen, causes acute gastroenteritis and mesenteric lymphadenitis, which are often accompanied by fever and abdominal pain. Although *Y. pseudotuberculosis* infections are distributed worldwide, little is known about their incidence and epidemiology in the United States. *Y. pseudotuberculosis* was first reported in the United States in 1938 and has rarely been identified since then (*1*). No outbreaks have been reported, and only 14 cases were documented from 1938 through 1973 (*2*). Although not reportable nationally, yersiniosis is a notifiable disease in all Foodborne Diseases Active Surveillance Network (FoodNet) sites. We describe the *Y. pseudotuberculosis* infections reported through FoodNet surveillance sites and compare these infections with those caused by the more commonly identified *Yersinia* species, *Y. enterocolitica*.

During 1996–2007, FoodNet conducted active surveillance for laboratory-confirmed *Yersinia* spp. infections (excluding *Y. pestis*) in Connecticut, Georgia, Maryland, Minnesota, New Mexico, Oregon, Tennessee, and selected counties in California, Colorado, and New York. All clinical laboratories in these areas were routinely contacted to ascertain cases. Demographic and outcome (e.g., hospitalization and death) information was collected for all cases. On the basis of the source of specimen collection, infections were categorized as invasive (isolated from cerebrospinal fluid, blood, or another normally sterile site) or noninvasive (isolated from urine, stool, or other site). Data were analyzed by using SAS version 9.2 (SAS Institute, Cary, NC, USA). Differences were evaluated by using χ^2^ and Fisher exact tests, and medians were compared by using the Wilcoxon rank-sum test. A 2-tailed p value of <0.05 was considered significant.

During 1996–2007, 1,903 *Yersinia* infections were reported in FoodNet sites. Of these, 1,471 (77%) had species information available. Most of the isolates were *Y. enterocolitica* (1,355; 92%); 18 (1%) *Y. pseudotuberculosis* infections were identified. The average annual incidence of *Y. pseudotuberculosis* infections was 0.04 cases per 1,000,000 persons. Most *Y. pseudotuberculosis* cases were reported from the western FoodNet areas of California (5 cases) and Oregon (5 cases).

The median age of persons with *Y. pseudotuberculosis* infection was 47 years (range 16–86 years), and 67% were male (Table). Of the 13 *Y. pseudotuberculosis* cases for which race was reported, 10 (77%) were in whites. Eight (44%) *Y. pseudotuberculosis* cases occurred in the winter months (December–February). Thirteen (72%) persons with *Y. pseudotuberculosis* infection required hospitalization; the median hospital stay was 9 days (range 2–35 days). Two deaths were reported, yielding a case-fatality rate of 11%. Twelve (67%) of the *Y. pseudotuberculosis* isolates were recovered from blood specimens, and only 1 isolate was recovered from stool.

**Table:** Comparison of *Yersinia pseudotuberculosis* and *Y. enterocolitica* infections, FoodNet, 1996–2007*

Characteristic	*Y. pseudotuberculosis*	*Y. enterocolitica*	p value
No. infections	18	1,355	
Annual average incidence† (range)	0.04 (0.00–0.10)	3.45 (0.77–7.87)	
Median patient age, y (range)	47 (16–86)	6 (0–94)	<0.0001
Male sex, no. (%) patients	12 (67)	672 (50)	0.1638
White race, no. (%) patients	10 (56)	480 (35)	0.0115
Western region of USA (CA, OR), no. (%)	10 (56)	308 (22)	0.0024
Winter season, no. (%)	8 (44)	536 (40)	0.8091
Invasive specimen collection site, no. (%)	12 (67)	106 (8)	<0.0001
Hospitalized, no. (%) patients	13 (72)	411 (30)	0.0003
Median hospitalization, d (range)	9 (2–35)	4 (0–107)	0.0118
Died, no. (%) patients	2 (11)	15 (1)	0.0248

*CA, California; OR, Oregon. †Cases per 1,000,000 persons.

In comparison, the average annual incidence of *Y. enterocolitic*a infections in FoodNet was 3.5 cases per 1,000,000 persons, and many of the cases occurred in the southern FoodNet site of Georgia (443 cases, 33%) (Table). Persons with *Y. enterocolitica* infection were significantly younger than those with *Y. pseudotuberculosis* infection (median age 6 years, p = 0.0002), and unlike *Y. pseudotuberculosis* infections, *Y. enterocolitica* infections were evenly distributed among male and female patients and among whites and blacks. Compared with those with *Y. enterocolitica* infection, persons with *Y. pseudotuberculosis* infection were more likely to be hospitalized (p = 0.0003), have longer hospital stays (p = 0.0118), die (p = 0. 0248), and have an isolate recovered from an invasive site (p<0.0001).

Most of the *Y. pseudotuberculosis* infections reported in FoodNet sites appeared to be severe and invasive. The rarity of diagnosed *Y. pseudotuberculosis* infections is consistent with earlier reports from North America (*3*,*4*), but this rarity remains unexplained. This rarity contrasts with the observation that cases and outbreaks are more common in other parts of the developed world, particularly in northern climes (*1*,^5^,*6*–*8*). The recent appearance of epizootic *Y. pseudotuberculosis* in farmed deer in the southern United States suggests that this could change (*9*).

The high proportion of *Y. pseudotuberculosis* cases that were diagnosed by blood culture suggests that less invasive *Y. pseudotuberculosis* infections are underrecognized in the United States. Diagnosis of *Yersinia* infections is difficult without specific culture, *Yersinia* is not routinely tested for in the United States, and isolation of the organism by culture may be difficult with standard media (*2*,*10*). Clinical diagnosis of *Y. pseudotuberculosis* infections can be challenging because physicians are not aware that *Y. pseudotuberculosis* is a potential cause of gastroenteritis (*10*). In the syndrome of pseudoappendicitis, the distinctive findings found by surgical exploration of severe mesenteric lymphadenitis can be suggestive, but diagnosis would require confirmation by culture of nodes or feces (*2*,*3*).

Unless the physician is both aware of *Y. pseudotuberculosis* as a cause of gastroenteritis and knows which diagnostic test to order, *Y. pseudotuberculosis* infections will go undiagnosed. Clinicians should consider *Y. pseudotuberculosis* as a cause of gastroenteritis and pseudoappendicitis and request appropriate microbiologic testing for patients with suspected cases. If more cases are identified in the United States, another investigation of *Y. pseudotuberculosis* might clarify the epidemiology of this infection.
